# Emotional Intelligence and Burnout Among Otorhinolaryngology–Head and Neck Surgery Residents

**DOI:** 10.3389/fpubh.2022.851408

**Published:** 2022-05-20

**Authors:** Abdulelah M. Sharaf, Isra H. Abdulla, Abdullah M. Alnatheer, Aghadir N. Alahmari, Omar A. Alwhibi, Ziyad Alabduljabbar, Hamzah Alhamzah, Feras M. Alkholaiwi

**Affiliations:** ^1^College of Medicine, Imam Mohammad Ibn Saud Islamic University, Riyadh, Saudi Arabia; ^2^College of Medicine, Al-Maarefa University, Riyadh, Saudi Arabia; ^3^Dr. Sulaiman Al Habib Medical Group, Riyadh, Saudi Arabia; ^4^Department of Otorhinolaryngology–Head and Neck Surgery, College of Medicine, Imam Mohammad Ibn Saud Islamic University, Riyadh, Saudi Arabia

**Keywords:** burnout, emotional intelligence, residents, Otorhinolaryngology-Head and Neck surgery, ENT

## Abstract

**Background:**

Burnout syndrome is common among surgical residents, negatively affecting their mental health, physical wellbeing, and work performance. We investigated the relationship between emotional intelligence (EI) and burnout among Otorhinolaryngology–Head and Neck surgery residents.

**Methods:**

This cross-sectional study examined 51 residents across different Otorhinolaryngology-Head and Neck surgery programs at various hospitals in Saudi Arabia using a survey conducted between January 2021 and March 2021. The questionnaire had different validated measurements of burnout and included the Trait EI Questionnaire–Short Form, Maslach Burnout Inventory–Human Services survey, and questions regarding demographics and job satisfaction.

**Results:**

Of all the residents, 17.6% had a high risk of burnout, 39.2% had emotional exhaustion (EE), 29.4% had depersonalization (DP), and 43.1% had a low sense of personal accomplishment (PA). A statistically significant negative association was observed between the total EI score and EE (r = −0.577, *p* < 0.001) and DP (r = −0.765, *p* < 0.001), indicating that higher total EI scores were associated with lower EE levels.

**Conclusions:**

Positive associations existed between high levels of EI, PA, and satisfaction with both surgical skills and specialty choice. Therefore, residency programs should use EI modifiers as tools to reduce the risk of burnout.

## Introduction

Burnout syndrome is defined as a physiological state characterized by emotional exhaustion (EE), depersonalization (DP), and a low sense of personal accomplishment (PA) (1, 2). Burnout can affect anyone regardless of their profession, but it mostly affects those with high-demand jobs, including healthcare providers (3). The Maslach Burnout Inventory (MBI) is a validated tool for the evaluation of burnout, and can be used to develop self-awareness about this issue (1).

The main causes of burnout are related to the nature of hospital work, since residents and physicians are expected to work long hours, can suffer from a lack of sleep, and are often on call. Healthcare providers can be affected by daily work requirements, such as discussing unfortunate events to patients or dealing with difficult patients (4). In 2015, a study conducted in Saudi Arabia found a higher incidence of burnout among Otorhinolaryngology-Head and Neck surgery (ORL-HNS) residents in the Saudi Board Training Program than in other board training programs (5). An additional study in 2011 found that ~41% of Otorhinolaryngology–Head and Neck surgeons presented with features of burnout (6).

Emotional intelligence (EI) is defined as “the awareness, control, and expression of one's emotions and the ability to handle interpersonal relationships judiciously and empathetically” (7, 8). In 1995, Goleman conceptualized EI as a set of competencies that can be trained and learned (9). Recently, the medical field has established the potential benefits of EI for the healthcare system, and there is growing interest in the importance of EI in medical training (10, 11). Moreover, higher EI in medical residents and internists is associated with a decreased incidence of burnout and a higher degree of job satisfaction (12). Few studies have examined the association between EI and burnout among surgical residents. A cohort study conducted in the Northeastern United States showed that high EI and positive work experiences were associated with a low incidence of burnout among general surgery residents (13). Other studies have suggested that EI may have a significant impact on a resident's ability to control the stress associated with medical training, and be useful for optimizing their wellness (14, 15). In this context, the present study aimed to investigate the relationship between EI and the individual components of burnout among ORL–HNS residents in Saudi Arabia.

## Materials and Methods

### Ethics Approval and Study Design

This study was conducted in accordance with the guidelines of the Declaration of Helsinki, and approved by the Institutional Review Board/ethics committee of Imam Mohammad Ibn Saud Islamic University, Riyadh, Saudi Arabia (No. 83-2021). Written informed consent was obtained from the participants to publish this paper. A cross-sectional study was conducted in a sample of ORL–HNS residents registered in different ORL–HNS programs across different hospitals, including university, military, national guard, security forces, and the Ministry of Health hospitals. The survey was conducted between January and March 2021, and data collectors distributed the survey questionnaires to the residents through social media as well as AirDrop (Apple Inc., Cupertino, California, USA). The participants are those residents from the second year to the fifth year in the ORL–HNS residency program and were informed of the purpose of the study before providing written informed consent.

#### Assessment Measures

The questionnaire used in this study included three main parts: demographics, the Trait EI Questionnaire–Short Form (TEIQue-SF), and the MBI–Human Services (MBI-HS) survey. Demographic information, such as sex and age, was assessed in Part A of the survey. Additional questions were included to assess job satisfaction, operating room role, and job salary, since these variables may affect EI. EI was assessed using the TEIQue-SF, an abbreviated version of the TEIQue. The TEIQue-SF includes 30 questions that assess four EI domains: wellbeing, self-control, sociability, and emotionality. All questions are graded on a scale from 1 (“completely disagree”) to 7 (“completely agree”). The overall global EI score is calculated by averaging the scores of all 30 items. The survey is scored using an online key (London Psychometric Laboratory). The TEIQue-SF provides a global trait EI score ranging from 1 to 7, and is calculated using the mean of the individual question scores. Factor scores also range from 1 to 7. The MBI-HS was used to assess the prevalence of various burnout domains among respondents. The MBI-HS targets healthcare services and includes 22 questions. The questions assess three domains of burnout: EE (nine questions), a sense of PA (eight questions), and DP (five questions). High scores on the DP and EE domains, and low scores on the PA domain, are associated with a higher risk of burnout. High EE is identified if the EE score is >26, while high DP is identified if the DP score is >12. A cut-off value of 32 is used to define the PA level. Thus, students with a PA score of <32 were classified as having a low sense of PA.

#### Statistical Analyses

Statistical analyses were performed using R software version 3.6.3 (R Foundation for Statistical Computing, Vienna, Austria). Continuous numerical variables, EI, and burnout dimensions were described as the mean ± standard deviation, and categorical data were summarized as numbers and percentages. Spearman correlations were used to assess the strength and magnitude of the linear association among the various EI dimensions and burnout. Multiple linear regression was used to model the multivariate effects of different variables as predictors of high EE, low PA, high DP, and EI as the main outcomes. Statistical significance was set at *p* < 0.05.

## Results

### Descriptive Statistics

The descriptive statistics of the study sample are provided in Table 1. The questionnaire was completed by Otorhinolaryngology residents (*n* = 51), the majority of whom were male (64.7%). The average age of the included residents was 27.9 ± 2.1 years. Approximately half (45.1%) of the respondents were married. Regarding residency year, 50% of the respondents were in their second, 15.7% were in their third, 17.6% were in their fourth, and 17.6% were in their fifth residency year. The average weight of the included residents was 71.9 ± 15.2 kg, with an average height of 169 ± 8.18 cm. The majority of study participants (68.6%) were non-smokers. A third (33.3%) of the residents reported not exercising at all, 25% reported exercising 2 days per week, and 23.5% reported exercising 3 days per week. The mean working period at the hospital was 13.8 ± 15.4 h per day, while the mean number of clinics attended per week was 3.12 ± 1.24. The average number of on-call events per month was 5.54 ± 1.92, while the average number of patients under daily care was 3 ± 2.5. A total of 10 (38.5%) students considered their families supportive, and a similar number thought their families were very supportive.

**Table 1:** Descriptive statistics of the study sample.

**Characteristics (*n* = 51)**	**Value**
Sex, *n* (%)	
Female	18 (35.3%)
Male	33 (64.7%)
Age (years), mean (SD)	27.9 (2.14)
Current level of residency program, *n* (%)	
R2	25 (49.0%)
R3	8 (15.7%)
R4	9 (17.6%)
R5	9 (17.6%)
Marital status, *n* (%)	
Married	23 (45.1%)
Not married	28 (54.9%)
Weight (kg), mean (SD)	71.9 (15.2)
Height (cm), mean (SD)	169 (8.18)
Smoker, *n* (%)	
No	35 (68.6%)
Yes	16 (31.4%)
Average number of days of exercise per week, *n* (%)	
0 days	17 (33.3%)
1 day	7 (13.7%)
2 days	12 (23.5%)
3 days	12 (23.5%)
4 days	2 (3.92%)
>4 days	1 (1.96%)
Hours of sleep per day, mean (SD)	6.29 (0.95)
Number of on-call events per month, mean (SD)	5.54 (1.92)
Hours per day of working in the hospital, mean (SD)	13.8 (15.4)
Number of clinics attended per week, mean (SD)	3.12 (1.24)
Number of surgeries performed per week, mean (SD)	2.81 (1.75)
Number of patients under daily care, mean (SD)	3.21 (2.50)
Spousal support, mean (SD)	
Not supportive	1 (3.85%)
Neutral	5 (19.2%)
Supportive	10 (38.5%)
Very supportive	10 (38.5%)

*SD, standard deviation*.

#### Assessments of Burnout

**Figure 1:**
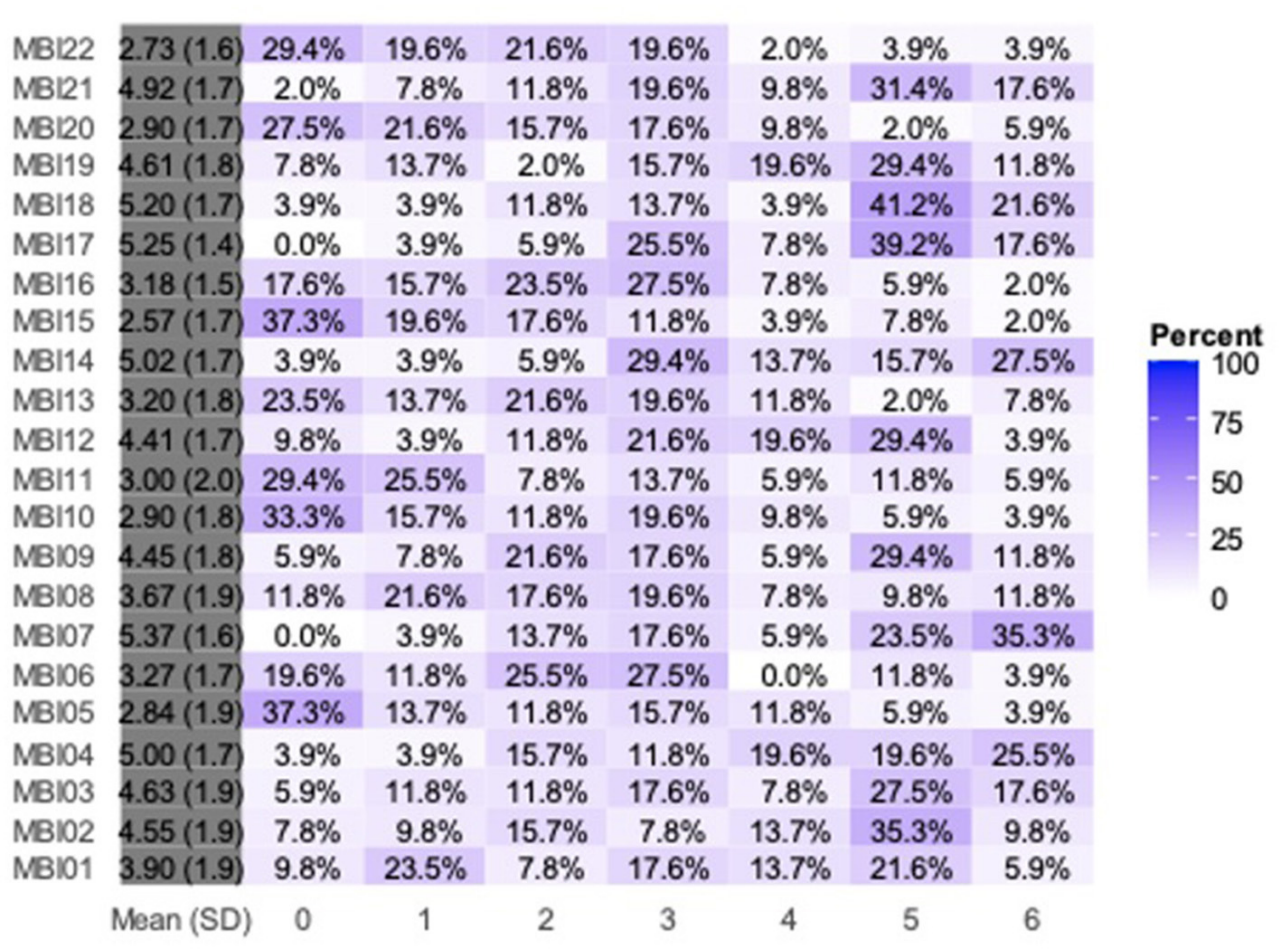
Responses to the Maslach Burnout Inventory–Human Services survey items. MBI, Maslach Burnout Inventory; SD, standard deviation.

**Figure 2:**
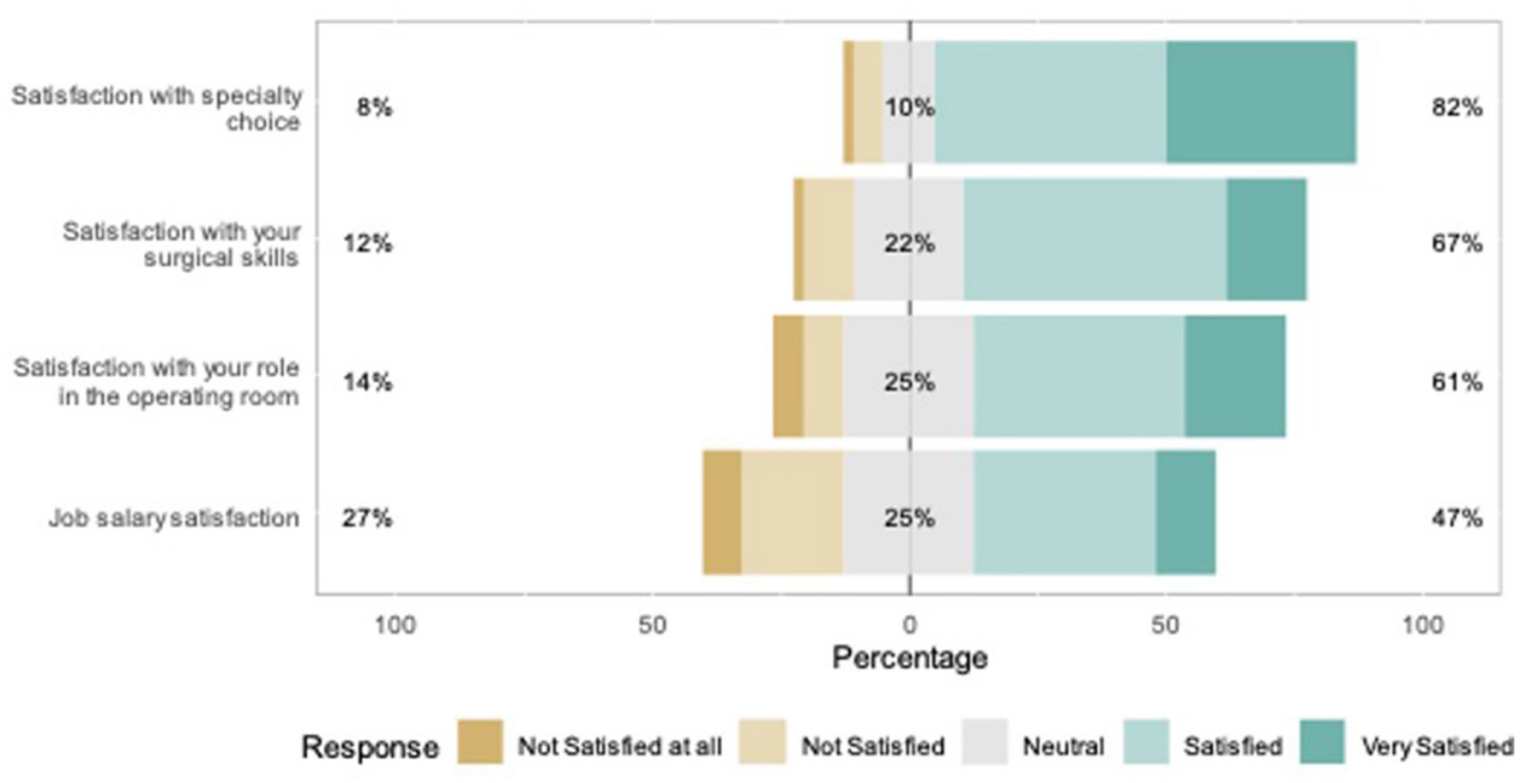
Satisfaction with various aspects of work.

Figure 1 shows the responses to the MBI-HS items. In total, 82% of the residents were satisfied with their specialty choice, while 8% were dissatisfied (Figure 2). Furthermore, 67% of the respondents were satisfied with their surgical skills, and 61% with their roles in the operating room. Less than half of the residents were satisfied with their job salaries. A high prevalence of EE among Otorhinolaryngology residents which was 39.2% (*n* = 20), while the prevalence of DP was 29.4% (*n* = 15). A low sense of PA was observed in 22 (43.1%) residents. Overall, nine (17.6%) participants had a high risk of burnout (Table 2).

**Table 2:** Correlations among emotional intelligence and burnout components.

	**TEI**	**Wellbeing**	**Self-control**	**Emotionality**	**Sociability**	**EE**	**DP**	**PA**
TEI		0.814^‡^	0.742^‡^	0.786^‡^	0.824^‡^	−0.577^‡^	−0.765^‡^	0.680^‡^
Wellbeing	0.814^‡^		0.508^‡^	0.446^†^	0.578^‡^	−0.567^‡^	−0.570^‡^	0.723^‡^
Self-control	0.742^‡^	0.508^‡^		0.472^‡^	0.507^‡^	−0.419^†^	−0.555^‡^	0.400^†^
Emotionality	0.786^‡^	0.446^†^	0.472^‡^		0.584^‡^	−0.230	−0.604^‡^	0.369^†^
Sociability	0.824^‡^	0.578^‡^	0.507^‡^	0.584^‡^		−0.577^‡^	−0.688^‡^	0.596^‡^
EE	−0.577^‡^	−0.567^‡^	−0.419^†^	−0.230	−0.577^‡^		0.590^‡^	−0.613^‡^
DP	−0.765^‡^	−0.570^‡^	−0.555^‡^	−0.604^‡^	−0.688^‡^	0.590^‡^		−0.637^‡^
PA	0.680^‡^	0.723^‡^	0.400^†^	0.369^†^	0.596^‡^	−0.613^‡^	−0.637^‡^	

Computed correlation used the Pearson method with listwise deletion. *p <0.05,

† *p <0.01*,

‡ *p <0.001*.

*DP, depersonalization; EE, emotional exhaustion; PA, personal accomplishment; TEI, total emotional intelligence*.

#### Correlations Between Burnout and Emotional Intelligence

Statistically significant negative associations were observed between total EI score and EE (r = −0.577, *p* < 0.001) and DP (r = −0.765, *p* < 0.001), indicating that a higher total EI score was associated with a lower EE. A higher PA score was significantly associated with a higher total EI score (r = 0.68, *p* < 0.001) (Table 3). A statistically significant negative correlation was observed between all EI and MBI-HS domains, except for the association between EE and emotionality (r = −0.23, *p* > 0.05). The positive correlation between EE and DP was statistically significant (r = 0.59, *p* < 0.001). Higher PA scores were significantly associated with lower DP scores (r = −0.637, *p* < 0.001) and lower EE (r = −0.613, *p* < 0.001). Statistically significant positive correlations were observed among all four EI domains, with values ranging from r = 0.446–0.824.

**Table 3:** Correlations among the various factors in the present study.

**Variable**	**EE**	**DP**	**PA**	**TEI**
Average number of days of exercise per week	−0.192	−0.157	0.216	0.447^†^
Average hours of sleep per day	−0.118	−0.277^*^	0.072	0.232
Average number of on-call events per month	0.356^*^	0.128	−0.125	−0.053
Average hours of working at the hospital per day	0.324^*^	0.002	−0.084	0.015
Average number of clinics attended per week	−0.188	−0.265	0.206	0.158
Average number of surgeries performed per week	−0.008	−0.141	0.401^†^	0.207
Average number of patients under daily care	0.237	0.142	−0.166	−0.109

*Computed correlation used Pearson method with listwise deletion*.

* *p <0.05*,

† *p <0.01*,

^‡^*p <0.001*.

*DP, depersonalization; EE, emotional exhaustion; PA, personal accomplishment; TEI, total emotional intelligence*.

The correlations between burnout and EI domains were all statistically significant (Table 2). Greater skill satisfaction was significantly associated with a higher total EI score (r = 0.40, *p* < 0.05). A statistically significant positive association was observed between satisfaction with specialty and the total EI score (r = 0.31, *p* < 0.05). Satisfaction with salary was not significantly associated with the total EI score (r = 0.26, *p* > 0.05). No statistically significant association was observed between satisfaction with the role in the operating room and total EI score (r = 0.21, *p* > 0.05). The number of patients under daily care and that of clinics attended per week were not significantly correlated with EI, EE, DP, or PA (all *p* > 0.05), as summarized in Table 3. The number of surgeries performed per week was significantly associated with the PA score (r = 0.401, *p* < 0.01). The average number of days of exercise per week showed a statistically significant positive correlation with the total EI score (r = 0.447, *p* < 0.001), whereas the average number of sleeping hours per day showed a statistically significant negative correlation with DP (r = −0.277, *p* < 0.05). EE showed statistically significant positive associations with the average number of on-call events per month (r = 0.356, *p* < 0.05) and average working hours at the hospital (r = 0.324, *p* < 0.05).

A statistically significant negative correlation was observed between satisfaction with salary and EE (r = −0.329, *p* < 0.05) (Table 3), suggesting that an increase in satisfaction with salary was associated with a decrease in EE score. Salary satisfaction was significantly positively correlated with the PA score (r = 0.37, *p* = 0.016), while there were statistically significant negative associations between satisfaction with specialty and EE (r = −0.463, *p* < 0.001), specialty and DP (r = −0.317, *p* < 0.05), and role in the operating room and EE (r = −0.414, *p* < 0.01). A statistically significant negative correlation was observed between satisfaction with skills and EE (r = −0.365, *p* < 0.01), as well as between satisfaction with salary and DP (r = −0.29, *p* < 0.05). Two important factors showed a positive association with PA: satisfaction with salary (r = 0.37, *p* < 0.01) and satisfaction with specialty choice (r = 0.305, *p* < 0.05).

### Linear Regression Analysis

Age, sex, and residency level were not significant predictors of the total EI score, and marital status was not a significant predictor of burnout. The average daily sleeping hours showed a statistically significant association with the total EI score (B = 0.026, *p* < 0.05), indicating that one extra sleeping hour is associated with a 0.44-unit increase in the average EI score. Exercise level also showed a statistically significant association with the EI score (B = 0.52, *p* < 0.05), indicating that the average EI score was 0.52 units higher in residents who exercised than in those who did not. None of the remaining sociodemographic characteristics were significant predictors of the EI score.

More regular physical exercise was a significant predictor of higher PA scores (B = 5.72, *p* < 0.05) (Tables 4, 5). Thus, the average PA score increased by 5.72 points in respondents who exercised at least once per week compared to those who did not exercise. None of the remaining sociodemographic factors were significant predictors of the EE, DP, or PA scores.

**Table 4:** Factors associated with the total emotional intelligence score.

**Predictors**	**Estimates**	**95% CI**	***p***
Sex: Male vs. Female	−0.21	−0.59–0.16	0.261
Age (1-year increase)	0.00	−0.11–0.12	0.931
Exercise: 1 day or more vs. Never	0.52	0.15–0.90	0.007^*^
Residency: R4 to R6 vs. R1 to R3	0.38	−0.12–0.87	0.131
Average daily sleeping hours: >6 vs. <6	0.44	0.05–0.82	0.026^*^
Smoker: Yes vs. No	0.00	−0.37–0.37	0.986
Marital status: Not married vs. Married	−0.24	−0.60–0.12	0.189

*Statistical analysis was performed using linear regression*.

* *p ≤ 0.05. CI, confidence interval; DP, depersonalization; EE, emotional exhaustion; PA, personal accomplishment*.

**Table 5:** Sociodemographic and residency-related factors associated with high emotional exhaustion, high depersonalization, and low personal accomplishment scores.

**Predictors**	**EE**		**DP**		**PA**	
	**Estimates (95% CI)**	***p***	**Estimates (95% CI)**	***p***	**Estimates (95% CI)**	***p***
(Intercept)	38.37 (−26.22–102.97)	0.237	9.06 (−24.02–42.15)	0.584	3.76 (−37.40–44.92)	0.855
Sex: Male vs. Female	−4.73 (−12.38–2.92)	0.219	2.58 (−1.34–6.50)	0.191	−3.04 (−7.91–1.84)	0.216
Age (1-year increase)	−0.07 (−2.41–2.27)	0.950	0.05 (−1.14–1.25)	0.928	0.88 (−0.61–2.37)	0.241
Exercise: 1 day or more vs. Never	−6.10 (−13.74–1.54)	0.115	−0.74 (−4.65–3.18)	0.706	5.72 (0.85–10.59)	0.022^*^
Residency: R4 to R6 vs. R1 to R3	−8.34 (−18.38–1.70)	0.101	−4.60 (−9.74–0.54)	0.078	0.40 (−5.99–6.80)	0.899
Average daily sleeping hours: >6 vs. <6	−0.03 (−7.85–7.79)	0.993	−3.59 (−7.59–0.42)	0.078	2.76 (−2.23–7.74)	0.271
Smoker: Yes vs. No	2.14 (−5.43–9.71)	0.572	−0.29 (−4.17–3.59)	0.880	3.60 (−1.23–8.42)	0.140
Marital status: Not married vs. Married	−2.92 (−10.37–4.53)	0.434	3.05 (−0.76–6.87)	0.114	−4.03 (−8.78–0.71)	0.094

*Statistical analysis was performed using linear regression*.

* *p ≤ 0.05. CI, confidence interval; DP, depersonalization; EE, emotional exhaustion; PA, personal accomplishment*.

## Discussion

In a study examining 684 Otorhinolaryngology residents in the United States, 76% had moderate burnout, and 10% had a high burnout rate. In our study, 17.6% of residents were at a high risk of burnout, despite an overall small sample size (*n* = 51). Residency training is arguably the most important and difficult period of a student's medical career. Physicians are required to improve their character and hone their skills as they become more knowledgeable in their fields, while carrying out a daily workload considered to be heavier than that of other higher level careers. These years of training can have a physical and psychological effect on residents. The high prevalence of burnout among residents in general, and surgical residency training in particular, is especially alarming (16). Despite a plethora of recent research on the magnitude of burnout and its consequences, research on the individual factors which predict burnout and how to deal with them is still lacking.

There are many causes of burnout that cannot be controlled or avoided, which makes those that can be controlled or improved more valuable. Studies on the significance of personality traits in predicting burnout have shown contradictory results. Therefore, personality traits may not have a consistently reliable predictive relationship with burnout (6, 13). EI, comprising wellbeing, self-control, sociability, and emotionality, was found to have a significant relationship with the propensity toward burnout among physicians previous studies (17). Therefore, EI assessment can be employed as a tool to better understand the individual experiences of residents regarding burnout, factors that make them more vulnerable, and protective factors against burnout. EI assessment might be a better strategic tool for lowering the risk of burnout than advising residents to seek mental health counseling, especially since the constituents of EI are modifiable (13).

In a meta-analysis of studies that measured burnout prevalence using the MBI, the international prevalence rate of burnout among surgical residents was calculated at 51%. In the United States, the prevalence rate of burnout among residents was 27–75%, depending on the specialty (18). When applying the strict scales of EE, DP, and PA of burnout syndrome, a study focused on surgical specialties found that 3% of the participants had burnout syndrome. Furthermore, when individually evaluating the subscales of burnout, the number of affected participants increased to 50%, with high EE or DP in one out of every three surgeons (19).

In our study, high EI scores were associated with lower EE scores, indicating that residents with high EI were better equipped to avoid EE. This represents the stress dimension of burnout syndrome that could lead to residents distancing themselves from work both cognitively and emotionally (20). High EE scores were positively associated with a higher average number of on-call events per month, and average working hours, but negatively associated with satisfaction in both salary and role in the operating theater. Thus, it is plausible that EE corresponds to the workload and decreases with improvement in efficacy and rewards. Similarly, DP, which represents the cynical aspect of burnout, was strongly associated with EE, and therefore is negatively correlated with EI. These findings confirm the importance of ensuring tolerable working hours for residents, since physical and mental exhaustion translates to EE. The need for integration of operating theater roles from the start of the residency program should be examined, as this also reflects lower EE levels.

The EI score was significantly positively correlated with a sense of PA, and satisfaction with both surgical skills and specialty choice. This could be explained by high EI increasing the ability to manage stress, while at the same time decreasing the chances of experiencing EE (20). A satisfactory job experience, sense of accomplishment, and satisfaction with their own skills may be easier to achieve for residents with higher EI traits. Some key findings from this study highlighted the importance of residents' lifestyles, suggesting that lifestyle modifications could help increase EI levels. This could further assist residents in dealing with different domains of burnout syndrome and reducing its associated risk. Average daily sleeping hours and the extent of physical exercise were both positively correlated with the total EI scores, and in the burnout domains, higher PA scores were significantly positively correlated with physical exercise.

Tolerable working hours, fair pay, and safe assignment of operating theater roles to residents have been shown to decrease EE, thereby enhancing EI. Physical exercise increases the sense of PA, which, combined with sufficient sleeping hours, reflects higher EI levels. All these aspects can be targeted to enhance EI and reduce the risk for burnout. Further research on EI factors and their relationship with burnout needs to be conducted across various specialties, and a detailed plan to protect residents against burnout is needed. Our study assessed the subjective perspectives of the residents. However, their work performance was not evaluated to confirm the effects of EI levels, making it a possible limitation. Moreover, the relatively small sample size might have contributed to a non-response bias.

## Conclusions

This study showed that surgical specialty residents with higher EI levels had a lower risk of burnout. Factors such as lifestyle modification can promote EI. Residency programs can benefit from EI measurements to estimate the risk of burnout and develop methods to prevent it.

## Data Availability Statement

The raw data supporting the conclusions of this article will be made available by the authors, without undue reservation.

## Ethics Statement

The studies involving human participants were reviewed and approved by Imam Mohammad Ibn Saud Islamic University (IMSIU). The patients/participants provided their written informed consent to participate in this study.

## Author Contributions

AS, IA, AMA, ANA, OA, ZA, and FM: conceptualization. AS, FA, IA, and ANA: methodology. ZA: software. AS and FA: formal analysis. FA and ZA: resources. AS: data curation. AS, IA, AMA, and ANA: writing–original draft preparation. AS, IA, AMA, ANA, and FA: writing–review and editing. FA: supervision. AS, AMA, and FA: project administration. All authors have read and agreed to the published version of the manuscript.

## Conflict of Interest

The authors declare that the research was conducted in the absence of any commercial or financial relationships that could be construed as a potential conflict of interest.

## Publisher's Note

All claims expressed in this article are solely those of the authors and do not necessarily represent those of their affiliated organizations, or those of the publisher, the editors and the reviewers. Any product that may be evaluated in this article, or claim that may be made by its manufacturer, is not guaranteed or endorsed by the publisher.
